# Molecular and Functional Characteristics of DNA Polymerase Beta-Like Enzymes From Trypanosomatids

**DOI:** 10.3389/fcimb.2021.670564

**Published:** 2021-08-05

**Authors:** Edio Maldonado, Sebastian Morales-Pison, Fabiola Urbina, Aldo Solari

**Affiliations:** ^1^Programa de Biología Celular y Molecular, Instituto de Ciencias Biomédicas, Facultad de Medicina, Universidad de Chile, Santiago, Chile; ^2^Laboratorio de Genética Molecular Humana, Programa de Genética Humana, Instituto de Ciencias Biomédicas, Facultad de Medicina, Universidad de Chile, Santiago, Chile

**Keywords:** *Trypanosoma cruzi*, DNA polymerase beta, kinetoplast DNA, trypanosomatids, BER

## Abstract

Trypanosomatids are a group of primitive unicellular eukaryotes that can cause diseases in plants, insects, animals, and humans. Kinetoplast genome integrity is key to trypanosomatid cell survival and viability. Kinetoplast DNA (kDNA) is usually under attack by reactive oxygen and nitric species (ROS and RNS), damaging the DNA, and the cells must remove and repair those oxidatively generated lesions in order to survive and proliferate. Base excision repair (BER) is a well-conserved pathway for DNA repair after base damage, single-base loss, and single-strand breaks, which can arise from ROS, RSN, environmental genotoxic agents, and UV irradiation. A powerful BER system has been described in the *T. cruzi* kinetoplast and it is mainly carried out by DNA polymerase β (pol β) and DNA polymerase β-PAK (pol β-PAK), which are kinetoplast-located in *T. cruzi* as well as in other trypanosomatids. Both pol β and pol β-PAK belong to the X-family of DNA polymerases (pol X family), perform BER in trypanosomatids, and display intrinsic 5-deoxyribose phosphate (dRP) lyase and DNA polymerase activities. However, only Pol β-PAK is able to carry out trans-lesion synthesis (TLS) across 8oxoG lesions. *T. cruzi* cells overexpressing pol β are more resistant to ROS and are also more efficient to repair 8oxoG compared to control cells. Pol β seems to play a role in kDNA replication, since it associates with kinetoplast antipodal sites in those development stages in trypanosomatids which are competent for cell replication. ROS treatment of cells induces the overexpression of pol β, indicating that plays a role in kDNA repair. In this review, we will summarize the main features of trypanosomatid minicircle kDNA replication and the biochemical characteristics of pol β-like enzymes and their involvement in BER and kDNA replication. We also summarize key structural features of trypanosomatid pol β compared to their mammalian (human) counterpart.

## Trypanosomatidae

Kinetoplastae, Trypanosomatidae, is a group of single flagellated parasites found in a wide range of geographic areas. All members in this group are exclusively parasitic and found primarily in insects, although a few genera have life cycles involving a secondary host, which might be vertebrates, and plants. Their presence can cause a considerable economic impact and several species can cause diseases in plants, insects, animals, and humans. The three main sicknesses caused by trypanosomatids in humans are African trypanosomiasis (caused by *Trypanosome brucei* and transmitted by the vector tsetse flies), Chagas disease (caused by *Trypanosome cruzi* and transmitted by triatomine bugs),and leishmaniasis (caused by several species of *Leishmania* transmitted by sandflies) (Nussbaum et al., 2010). Three parasite genera are dixenous (two hosts in the life cycle), *Leishmania, Phytomonas*, and *Trypanosoma*, while all the rest are monoxenous (one host in the life cycle) (Overath et al., 2001). Trypanosoma species can employ two different forms of development inside the invertebrate host vector. The Salivary species are characterized by parasites that can develop in the initial portion of the invertebrate digestive system and transmitted to the vertebrate host through the insect bite (Stevens and Gibson, 1990; Haag et al., 1998). Meanwhile, the Stercorary species are characterized by their development in the invertebrate posterior region of the digestive system and transmitted to the vertebrate host through urine-feces excretion (Stevens and Gibson, 1990; Haag et al., 1998).

Trypanosomatids can cause agricultural and non-agricultural economical losses. The honeybee *Apis mellifera* can pollinate crop fields and fruit trees, however, the trypanosomatid pathogen *Crithidia mellificae* can infect the honeybees, causing economical losses in terms of honey production and the agriculture for food production (Ravoet et al., 2013; Runckel et al., 2014).

*Phytomonas* is a ubiquitous and diverse genus of plant parasites distributed in a wide range of tropical and subtropical geographic areas (Sturm et al., 2007; Votýpka et al., 2010). *Phytomonas ssp* were first described from the latex of the Mediterranean spurge *Euphorbia pilulifera* (Lafont, 1909). They can grow and develop in latex tubes, phloem, fruit sap, seeds, and nectar of many plant families (Sturm et al., 2007; Votýpka et al., 2010). Currently, the *Phytomonas* genus includes more than two hundred species, which can colonize more than twenty plant families and are pathogenic in the phloem of the coffee tree, coconut palms, and oil palms (Camargo, 1999; Dollet et al., 2000). In those plants, this can cause lethal diseases and destruction of plantations in Central and South America*. Phytomonas* are transmitted by the nocturne coreid spurge bug *Dicranocephalus agilis*, which is the natural vector.

*Leishmanias* is a very diverse genus distributed worldwide, and over 20 different species have different animal hosts, which are transmitted by over 90 sandfly insect vectors. Leishmaniasis is one of the seven most important neglected tropical and subtropical diseases, which is found in all continents, apart from Oceania (Torres-Guerrero et al., 2017). It is endemic to Asia, Africa, the Mediterranean region, and the Americas. *Leishmania* species can generate three different forms of disease that are cutaneous, mucocutaneous, and visceral with a potentially fatal outcome (Torres-Guerrero et al., 2017). Diagnosis (case finding) and treatment of leishmaniasis can cost $11-22 as measured by the dis-ability-adjusted life-years (DALY) measure averted in developing countries (Conteh et al., 2010). Parasite identification is clinically relevant since it is known that there exists a link between the Leishmania species and disease severity, which can influence the treatment outcome (Arevalo et al., 2007).

The *T. brucei* causes African trypanosomiasis or sleeping sickness, which is restricted to tropical Africa, mainly in rural areas, where wild and domestic animals act as a reservoir of the disease. The parasite develops in the salivary gland of the tsetse fly and it is transmitted by the insect bite (Nussbaum et al., 2010). Diagnosis and treatment of the African trypanosomiasis cost $12-24 per DALY averted in developing countries (Conteh et al., 2010). This treatment cost is mainly due to private drug donations or preferentially priced medicaments.

Chagas disease or American trypanosomiasis is caused by *T. cruzi* and has two phases (Bern, 2015). The initial acute phase can last several weeks and then some patients can develop a chronic phase which might continue for decades and can generate mega-organ syndrome, cardiomyopathy, and sudden death (Bern, 2015). This disease remains as the most important parasitic disease in the Western hemisphere with an estimated disease burden, as measured by DALY, that is 7.5 times bigger than malaria (Lee et al., 2013)*. T. cruzi* is transmitted to vertebrate hosts by Stercorary triatomine bugs, which are distributed mainly in rural areas from South USA to Argentina and North Chile (Nussbaum et al., 2010).

It is thought that trypanosomatids have a single origin, exclusively as insect-borne parasites, and then become digenic when vertebrates appeared in the Mesozoic era about 230 million years ago (Hamilton et al., 2004). Despite being evolutionarily primitives and mainly asexually reproduced, however, they have developed genetic recombination mechanisms as evolution driving forces. After the fusion of two diploid cells (a kind of asexual mating) and later nuclear erosion, the tetraploid cell eventually tends to return to the diploid stage after genetic exchanging, as described for *T. cruzi* hybrid strains (Westenberger et al., 2005). It is currently accepted that reproduction involving asexual meiosis can explain *T. brucei* variability, a process that occurs in the salivary gland of the insect vector (Gibson, 1990). This parasexual pathway is also observed in axenic cultures of *Leishmania*, although seems to be unlikely or rare in nature (Chogas, 1986; Cruz et al., 1993; Laurent et al., 2007). This genetic exchange mechanism has been also observed in some fungi (Heitman et al., 2014). On the other hand, in other parasites, such as *Plasmodium falciparum* (which causes malaria), sexual reproduction is an obligatory part of the life cycle, and mating must occur during every transmission cycle through the mosquito vector (Rono et al., 2018).

Trypanosomatids can respond rapidly and efficiently to quick environmental changes, though cannot regulate gene expression at the transcriptional level as higher eukaryotes do (De Pablos et al., 2016). However, they can mainly regulate gene product functions at the post-transcription or post-translational levels (De Pablos et al., 2016). Also, *Leishmania* under conditions of severe stress can amplify single-copy genes to obtain a high amount of those gene products (De Pablos et al., 2016). In experimentally induced drug-resistant *Leishmania* strains, it is common to find circular episomes, which are the result of the amplification of short chromosomal regions containing key enzymes involved in DNA nucleotide metabolism and overexpression (by gene amplification) of specific genes involved in drug resistance, such as against antimonial drugs, which are used to treat leishmaniasis (De Pablos et al., 2016). Trypanosomatids must respond to extracellular and intracellular signals as they should adapt quickly to new environments inside their various hosts. Transcriptional responses are absent in trypanosomatids as their transcriptional units are polycistronic and their promoters do not contain gene regulatory sequences as in higher eukaryotes (De Pablos et al., 2016). Therefore, the regulation must be through mRNA processing, mRNA translation, mRNA stability, protein stability, and modification. Interestingly, after heat shock in *T. brucei*, changes in mRNA compartmentalization are observed (Minia et al., 2016). The untranslated mRNA of the zinc-finger protein ZC3H11 is present in the cytosol, however, after heat shock, it moves to a polysomal fraction and escapes sequestration into the granules and the mRNAs bound for ZC3H11 remained in the polysomal fraction to be translated (Minia et al., 2016). The function of ZC3H11 has been shown to be regulated by protein kinases. Interestingly, another example of rapid protein regulation to oxidative stress is the *T. cruzi* pol β, which can be overexpressed and quickly activated by protein kinases after acute exposition to hydrogen peroxide (Rojas et al., 2018).

Considering the importance of trypanosomatids as pathogens of humans, animals, plants, and insects is important to know the basic biological process that controls their complex life cycle. Trypanosomatids are early-branching eukaryotes, and their primitive lineage has revealed unusual biological features, and probably their most notable characteristic is the mitochondrial DNA, named kinetoplast DNA (kDNA). Therefore, we will describe the kDNA repair and minicircle kDNA replication processes of those parasites with an emphasis on the function and involvement of pol β-like enzymes in DNA repair and replication.

## Base Excision Repair

The eukaryotic cell possesses mitochondrial and nuclear genomes, which can replicate and accumulate mutations. Nuclear damaged DNA is repaired by multiple, often overlapping, DNA repair systems, however, mitochondrial DNA has a more restricted repair system. Since reactive oxygen species (mtROS) are produced in the mitochondria, the genome is altered; therefore, this organelle must possess a DNA repair system to deal with oxidative damage. The DNA repair systems are essential to maintain genome integrity and can therefore avoid mutations to ensure cell survival. In the nucleus, DNA lesions are frequently generated; abasic sites and DNA strand breaks that are oxidized, deaminated, and alkylated are repaired mainly by the BER system (for a comprehensive review see references 28 and 29). The BER system has been extensively studied in mammalian cells and starts with a DNA glycosylase that removes the damaged base, producing an apurinic/apyrimidinic site (AP site) followed by an incision of the phosphodiester backbone 5′ to the abasic site by an AP endonuclease (Krokan and Bjørås, 2013; Beard et al., 2019; Mullins et al., 2019). This step leaves a single-nucleotide gap with 3′-hydroxyl and 5′-deoxyribose phosphate at the gap margins. Afterward, pol β incorporates the missing nucleotide according to the template instructions and removes the 5’- deoxyribose-5- phosphate (intrinsic dRP lyase activity), and the chain is sealed by a DNA ligase (Krokan and Bjørås, 2013; Beard et al., 2019). In the short-patch pathway (SP-BER) a unique nucleotide is inserted by pol β, but in the long-patch pathway (LP-BER) two or more nucleotides can be incorporated by pol β or pol λ (Garcıa-Dıaz et al., 2001; Braithwaite et al., 2005; Braithwaite et al., 2005; Krokan and Bjørås, 2013). Both the SP-and LP-BER are sub-pathways of BER, and they have common steps. One of the most common lesions caused by ROS is the formation of 7,8-dihidro-8-oxoguanine lesions (8-oxoG), which if left unrepaired can lead to mutagenic GC-TA transversions during cell replication (Krokan and Bjørås, 2013; Beard et al., 2019). In the nuclei of mammalian cells, most of the DNA oxidative damage is repaired by BER, and pol β is the principal polymerase involved in this process (Krokan and Bjørås, 2013; Beard et al., 2019). However, pol λ also plays a minor role in BER during the repair of oxidative DNA damage and possesses a domain with dRP lyase activity (Garcıa-Dıaz et al., 2001; Braithwaite et al., 2005; Braithwaite et al., 2005). Mammalian pol β is a small 335-amino-acid-residues-long polypeptide possessing two domains that correspond to an N-terminal 90-amino-acid-residues-long domain (8 kDa amino-terminal domain) and a 265-amino-acid-residues-long C-terminal domain (31 kDa carboxy-terminal domain) (Beard and Wilson, 2019). The amino-terminal domain has intrinsic lyase activity and can remove the 5’deoxyribose phosphate, which is left after incision by an apurinic/apyrimidinic endonuclease (AP) in the course of BER. The C-terminal domain has DNA polymerase activity and is involved in template-directed gap-filling of DNA. Although it was thought that mammalian pol β was located exclusively into the nucleus, it has been recently found that pol β is associated with mouse and human mitochondria of brain and kidney cells, indicating a role of this enzyme in mitochondrial DNA repair (Sykora et al., 2017; Prasad et al., 2017; Kaufman and Van Houten, 2017). The size of pol β detected in mitochondria and nuclei is the same, indicating that both are identical proteins.

## DNA Polymerase X-Family

The pol X family can be found in all life kingdoms and are phylogenetically conserved. In mammals, the pol X family is comprised of pol β, pol μ, pol λ, and terminal deoxynucleotidyl transferase (TdT) (Bienstock et al., 2014). Plants, fungi, and simpler organisms (bacterias) possess only one or two family members, and a pol X member has been also found in the African swine fever virus (Bienstock et al., 2014). Surprisingly, in some metazoan organisms, such as *Caenorhabditis elegans* and *Drosophila melanogaster*, pol X family members are not present. Pol X family members are mainly involved in DNA repair pathways, such as BER, nonhomologous end joining (NHEJ), and V(D)J recombination. Typically, the members of this family show low fidelity; contrastingly, family A and B members are highly accurate and function in DNA replication and repair of replication errors. In mammals, the pol X family are rather small proteins ranging from 35-75 kDa, and they share a common domain structure, which is a BRCT domain (BRCA1 C-terminal) at the N-terminus (with exception of pol β), a Ser/Pro-rich domain (exclusively in pol λ), a middle lyase domain, and a C-terminal polymerase domain with three subdomains: DNA-binding (D), catalytic (C), and nucleotide binding (N) (Bienstock et al., 2014). The BRCT domain is essential for interactions with functional partner proteins of the NHEJ system, such as Ku antigen, DNA ligase IV, and XRCC4 in vertebrates. The lyase domain is involved in dRP lyase activity and is only active in pol β and pol λ, which are involved in BER (Bienstock et al., 2014). On the other hand, the polymerase domain is involved in DNA synthesis.

Fungi, such as *Schizosaccaromyces pombe*, contain a pol X (SpPol4) closely related to mammalian pol μ, whereas *Saccaromyces cerevisiae* pol X (Scpol4) is related to mammalian pol λ (Bienstock et al., 2014). On the other hand, plants have only a pol λ orthologue. Trypanosomatids are unusual since they have two pol β-like enzymes, which are named pol β and pol β-PAK. Those polymerases in most of the trypanosomatids are mitochondrial, although in *Leishmania* pol β is nuclear as well as in higher eukaryotes. Interestingly, trypanosomatid pol β is related to vertebrate pol β, as they share the same ancestor gene (Bienstock et al., 2014). Both, pol β and pol β-PAK are involved in kDNA replication and kDNA repair. Therefore, we will focus mainly on the biological functions of trypanosomatid pol β and pol β-PAK.

## Kinetoplast DNA Replication

**Figure 1:**
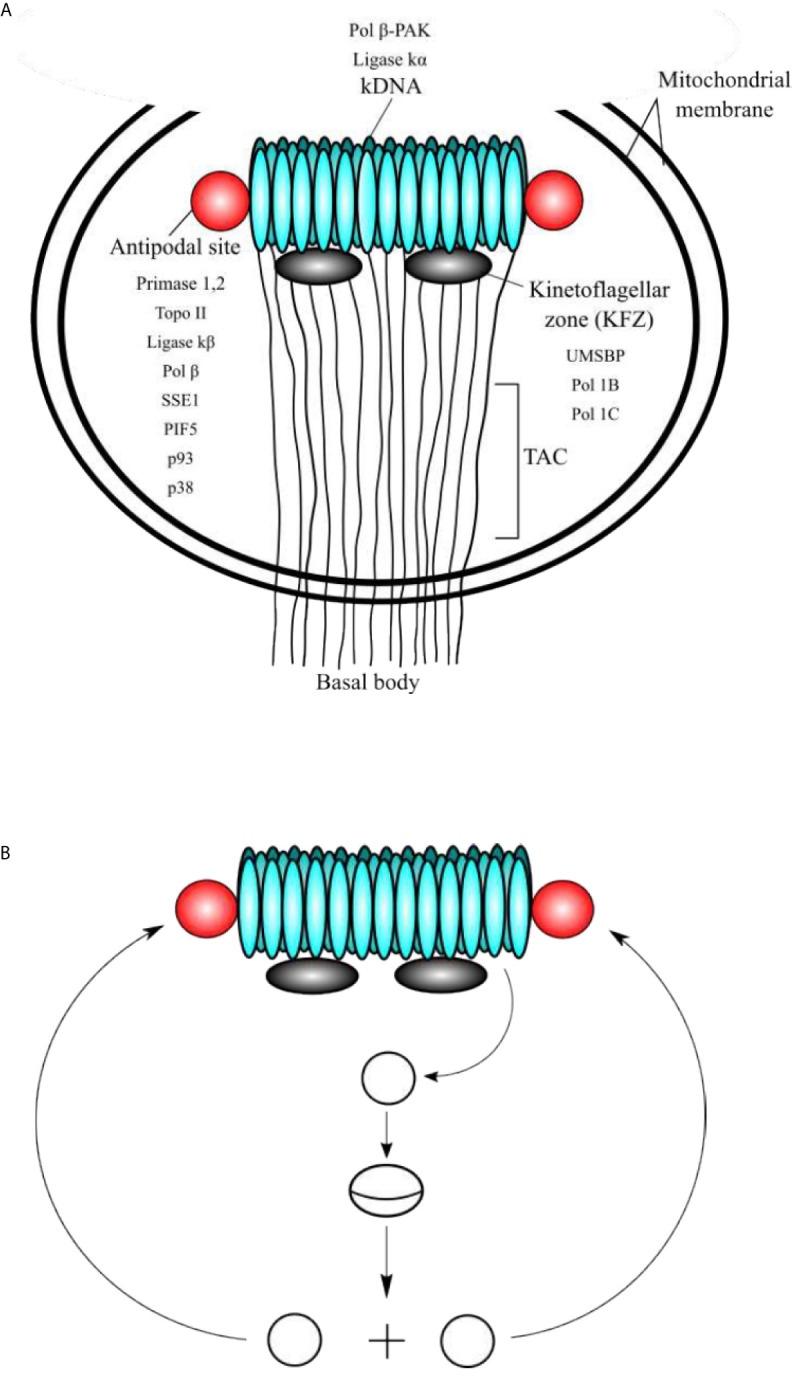
**(A)** Schematic representation of the domains of kinetoplast. **(A)** The kinetoplast domains include the kDNA disc, the antipodal sites, the kinetoflagellar zone (KFZ), the tripartite attachment complex (TAC), and the basal body, which is located outside of the mitochondrial membrane. The locations of critical proteins involved in minicircle kDNA replication are shown. **(B)** At the beginning of replication, the minicircles are released into the KFZ from the network through decatenation by a type II DNA topoisomerase (yet to be identified), where proteins that initiate replication locate. Replication proceeds as a theta structure and the newly replicated sister minicircles migrate to the antipodal sites. The next steps of kDNA replication occur at the antipodal sites, which contain a set of proteins involved in kDNA replication. Minicircles are attached to the network by topo II, where RNA primers are removed by SSE1/PIF5 and gaps are filled by pol β and nicks are sealed ligase κβ. Those minicircles that contain at least one gap are repaired at the kDNA disc by pol β-PAK and ligase κα.

**Figure 2:**
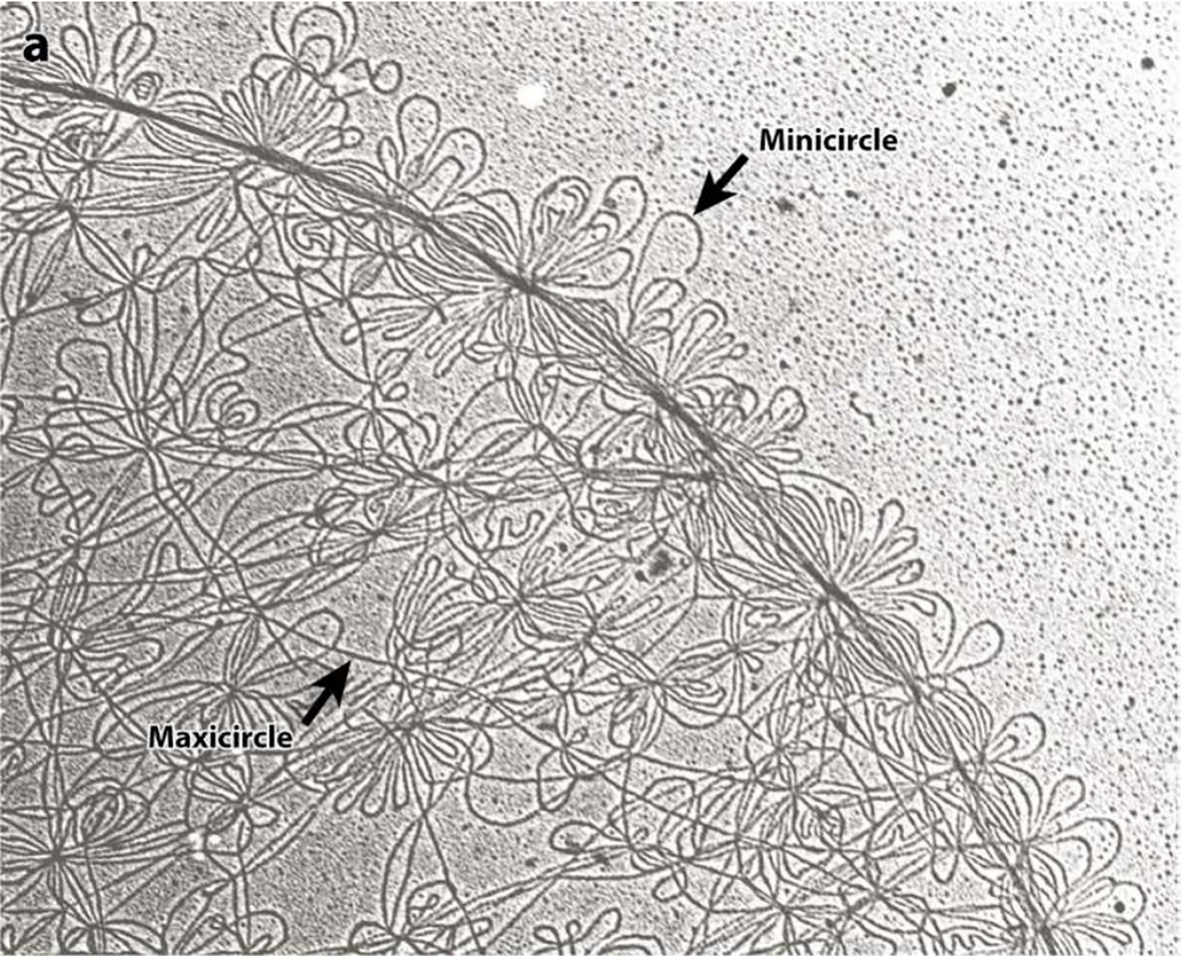
Electron microscopy photographs of the isolated kDNA network from *C. fasciculata* show the minicircles (small DNA loops) and maxicircles (long strands). Both, minicircles and maxicircles are indicated by the arrows. This figure was reproduced from reference 40. Each minicircle has a contour length of 0.7 microns.

Trypanosomatids contain a single and large mitochondrion, with a specialized region of the mitochondria containing an unusual DNA, which is named kinetoplast and is a characteristic structure of the Kinetoplastida order. The kinetoplast contains several discrete domains and those are described in **Figure 1A** (Cavalcanti and de Souza, 2018). The kDNA is a network of circular DNA, consisting of thousands of interlocked DNA circles. Those DNA circles are of two types, the maxicircles (20-40 Kb, depending on the species) and the minicircles, which are 1.0-2.5 Kb in size and depend upon the species (Jensen and Englund, 2012; Botero et al., 2018; Cavalcanti and de Souza, 2018). The minicircles and maxicircles can be observed in **Figure 2**. There are only a few dozen identical copies of maxicircles, which are analogous to the mitochondrial DNA of higher eukaryotes, and several thousand minicircles, which can differ in size and sequence between species. The DNA maxicircles encode the mitochondrial gene products, whereas the DNA minicircle encodes guide RNAs (gRNAs), which contain the genetic information to edit mitochondrial RNA transcripts (Benne, 1990; Jensen and Englund, 2012).

The replication mechanism of the kDNA is not well understood yet, however, the minicircle replication has been more studied than the maxicircle replication and is better understood. It is important to note that the proteins involved in this process have different locations within the kinetoplast with respect to the kDNA disk (see **Figure 1A**). The location of the proteins and enzymes involved in kDNA replication indicates that the events of replication of the kDNA are spatially regulated, and they proceed in an orderly fashion (Jensen and Englund, 2012). Minicircles contain a conserved region, with three highly conserved sequence blocks (CSB), namely, CBS-1, CBS-2, and CBS-3 (Ray, 1989). The sequences of those regions in *T. cruzi* are CBS-1 (AGGGGCGTTC; 10 bp), CSB-2 (CCCCGTAC; 8 bp), and CBS-3 (GGGGTTGGTGTA; 12 bp) (Botero et al., 2018). Both CBS-1 and CBS-2 present lower interspecies homology, however, CBS-3 is highly conserved in most of the trypanosomatids, also named Universal Minicircle Sequence (UMS), and is part of the minicircle replication origin (Ray, 1989; Jensen and Englund, 2012; Botero et al., 2018). The number of UMS in each minicircle depends on the species and the *T. cruzi* minicircles contain four UMS, whereas the *T. brucei* minicircles contain a single UMS (Botero et al., 2018). UMS is the specific DNA binding site for the protein UMS binding protein (UMSBP), which is involved in kDNA replication and segregation (Abeliovich et al., 1993; Milman et al., 2007). This protein has been well studied in *Crithidia fasciculata* and is also present in other trypanosomatids such as *T. brucei* and *Leishmania donovani* (Milman et al., 2007; Jensen and Englund, 2012; Englund, 2014). The importance of the UMSBP has been demonstrated in *T. brucei* in which the knockdown of both UMSBP genes affects the kDNA minicircles replication initiation, segregation of the daughter networks, and also blocks nuclear division (Milman et al., 2007; Englund, 2014). UMSBP is a single-stranded sequence-specific DNA binding protein that can bind to the UMS and an octameric sequence conserved at the replication origin of *C. fasciculata* kDNA minicircles (Tzfati et al., 1995; Singh et al., 2016). UMSBP is a small conserved protein that contains five tandemly arranged zinc knuckle motifs. Each motif forms a compact zinc finger containing the core motif CysX2CysX4HisX4Cys (X represents any amino acid). UMSBP orthologues have been described in *T. brucei* (TbUMSBP1) (Wang et al., 2000) and in *T. cruzi* (PDZ5; TcUMSBP1) (Coelho et al., 2003) and they can be found in most of the trypanonosomatid genomes listed at the NCBI Genbank by using the BLASTP bioinformatic tool.

Moreover, *C. fasciculata* UMSBP binding to UMS is regulated by the redox potential. It has been shown that UMSBP activity cycles throughout the trypanosomatid cell cycle and the activity tightly correlates with the UMSBP redox state (Sela and Shlomai, 2009). The oxidation of UMSBP can result in dimerization with inhibition of its binding activity to the UMS, while the reduction of UMSBP can produce monomers that can easily bind to the UMS (Sela and Shlomai, 2009). However, the binding of UMSBP to the replication origin is not regulated by the protein oligomerization state. It has been shown that loss of UMSBP DNA-binding activity by oxidation is a consequence of intra-molecular generation of disulfide bonds, and this event does not affect oligomerization (Sela and Shlomai, 2009). UMSBP oligomerization occurs in zinc-depleted unfolded zinc finger domains, but the zinc presence is essential for UMSBP binding to the replication origin of kDNA minicircles (Sela and Shlomai, 2009). This indicates that the binding of USMBP to the UMS depends on intact properly folded zinc finger domains. The trypanothione-dependent tryparedoxin is able to activate the binding of UMSBP to the UMS DNA sequence, indicating that binding of the UMSBP at the replication origin of the kDNA minicircles is regulated by a redox mechanism (Sela and Shlomai, 2009).

**Figure 3:**
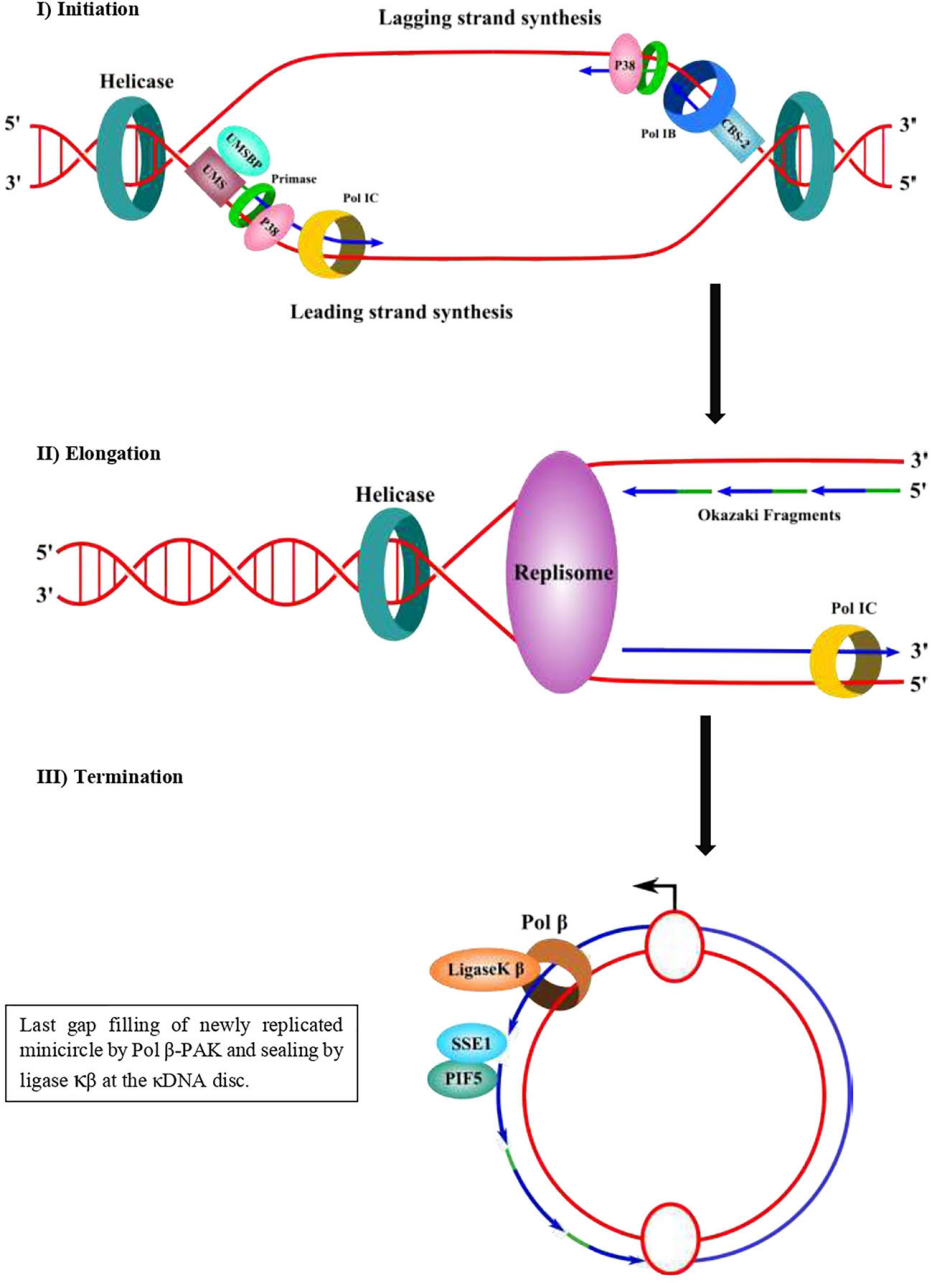
Proposed molecular mechanism of minicircle kDNA replication. In a first step (initiation), UMSBP binds to the CBS-3 (UMS) at the replication origin and recruits a helicase/p38 complex, which in a complex with a DNA helicase can open the replication origin using ATP hydrolysis and recruits a primase/pol I complex. The leading strand synthesis starts near the UMS and the lagging strand synthesis starts near CBS-2 (former hexamer). CBS-1 lies downstream and close to CBS-2. In a second step (elongation), a replisome elongates the DNA daughter strands of both the leading strand (continuous) and the lagging strand as Okazaki fragments. The lagging strand can be synthesized by pol IB and the leading strand could be replicated either by pol IB or pol IC. It is not clear yet whether pol IB synthetizes both strands and whether another pol I (pol IC) could synthesize the other strand. A DNA helicase should separate the DNA strands and DNA topoisomerase should manage the supercoiling produced during the movement of the replisome. In a third step (termination) and after synthesis of both strands, the sister minicircles migrates to the antipodal sites and primers from Okazaki fragments are eliminated by SSE1/PIF5, followed by gap-filling by pol β and sealed by ligase κβ and then attached to the network periphery by topo II. Some of the newly replicated minicircles might contain one or two gaps and they are gap-filled by pol β-PAK and sealed by ligase κα to terminate the replication process. Primers of the Okazaki fragments are shown in green. Newly synthesized DNA is shown in blue.

Prior to the replication, the minicircles are released from the network into the kinetoplast flagellar zone (KFZ) by a type II DNA topoisomerase and the UMSBP should bind to the UMS (CBS-3) of the minicircle replication origin and start the recruitment of the other protein components needed for replication to proceed. The UMSBP locates at two sites in the KFZ whereupon minicircle replication begins (Benne, 1990; Jensen and Englund, 2012; Englund, 2014). It is still unknown the number of components involved in the replication of kDNA minicircles, but this might be 50 or 100, due to the complexity of the process since the kDNA network has an unusual organization, which imposes constraints to the process. The knowledge of the functions of the proteins involved in the process can lead to a partial model of kDNA replication as proposed by Englund and colleagues (Benne, 1990; Jensen and Englund, 2012; Englund, 2014) and detailed here (**Figures 1A, B**). Once the UMSPB is bound to the kDNA replication origin (UMS) of the minicircles, a Pol I-like and a DNA primase should bind to the replication origin, recruited by UMSBP (**Figure 3**) and perhaps other auxiliary proteins as well, to start the synthesis of the daughter strands (Benne, 1990; Jensen and Englund, 2012; Botero et al., 2018). The replication of the leading strand starts near the UMS, and the replication of the lagging strand begins near the CBS-2 block. The CBS-1 block lies downstream and close to CBS-2. The replicative DNA polymerase seems to be pol IB (and perhaps pol IC in the leading strand), which could replicate both the leading and the lagging strand of the minicircle (Bruhn et al., 2010). However, the involvement of another pol I-like, such as pol IC, cannot be ruled out, and it might be possible that pol IB replicates the lagging strand and pol IC could replicate the leading strand as proposed in **Figure 3**. The replication proceeds unidirectionally from the UMS in a theta structure intermediate, resulting in a single gap in the leading strand and multiple gaps between Okazaki fragments in the lagging strand. The sister minicircles are believed to migrate to the antipodal sites, where RNA primers must be removed from the Okazaki fragments by PIF5 a DNA helicase that functions in primer removal from Okazaki fragments together with structure-specific endonuclease 1 (SSE1), an enzyme with RNAase H activity (Jensen and Englund, 2012; Englund, 2014). Thereafter, most of the gaps are filled by pol β to be sealed by DNA ligase κβ (ligase κβ), and the newly replicated minicircle is attached to the periphery of the kDNA network by DNA topoisomerase II (topo II) (Jensen and Englund, 2012). However, one or two gaps are maintained on some of the newly replicated minicircles after attachment to the network and they are probably filled by pol β-PAK” and sealed by DNA ligase κα, most likely at the kDNA disk, where pol β-PAK and DNA ligase κα (ligase κα) locate (Jensen and Englund, 2012). After the whole process is finished, the progeny networks are segregated into the daughter cells. The supercoiling produced during minicircle DNA replication should be managed by mitochondrial DNA topoisomerase IA to allow the replisome to progress and for proper segregation of the progeny molecules (Scocca and Shapiro, 2008; Jensen and Englund, 2012). The current model of minicircle kDNA replication is displayed in **Figures 1A, B**, and we propose a molecular model of minicircle kDNA replication in **Figure 3**. The main proteins that have been identified and that participate in minicircle kDNA replication are listed in **Table 1**.

**Table 1:** Main proteins involved in minicircle kDNA replication that have been identified.

Name	Accesion Genbank	Function
Primase 2	RHW74362^*^	Minicircle primase
Topo II	CAA42182	Minicircle attachment to kDNA disc
Ligase κβ	AAQ88427	Gap sealing
Pol β	AAA68599	Gap filling
p93	RHW73544^*^	kDNA replication
p38	AAO39843	Binds replication origin
UMSBP	AAC32813	Binds UMS
Pol IB	AAM81963^*^	kDNA synthesis
Pol IC	AAM81964^*^	kDNA synthesis
Pol β-PAK	AAQ516190^*^	Gap filling
Ligase κα	AAY22182	Gap sealing
SSE1	AF124228	RNA primer removal
PIF5	RHW71036^*^	Helicase for RNA primer removal

For each identified protein, name, accession number, and function are indicated. Those proteins labeled with * have been identified from Trypanosoma brucei and the rest from Crithidia fasciculata.

## Molecular Characteristics of Trypanosomatid pol β

As stated earlier, trypanosomatids have two DNA pol β-like enzymes. Pol β locates at the mitochondria, together with another DNA pol β-like polymerase, which is named pol β-PAK. Both DNA polymerases function in the BER pathway of the kDNA repair of oxidative lesions and in replication of the kDNA minicircles.

The pol β from *C. fasciculata* was the first trypanosomatid mitochondrial DNA polymerase of this family to be purified and its encoding gene was cloned (Torri et al., 1994; Torri and Englund, 1995). Later, by searching the genome database of *T. brucei*, two genes encoding two pol β-like enzymes were discovered (Saxowsky et al., 2003). One is the homolog of *C. fasciculata* pol β and the other was distantly related, and it was named pol β-PAK. Both enzymes are mitochondrial, however, their location into the kinetoplast is different. Pol β locates at the antipodal sites, whereas pol β-PAK locates at the kDNA disk (Saxowsky et al., 2003). Pol β-PAK from *T. brucei* has unusual structural features, such as a long N-terminal domain, which is rich in Proline, Alanine, and Lysine residues (PAK domain); however, this domain is absent in homologues from *T. cruzi* and *L. infantum*, although they are also called pol β-PAK.

On the other hand, *T. cruzi* pol β was first purified by our group from *T. cruzi* epimastigotes cell extracts. The major polypeptide of the purified fractions had a 50 kDa molecular weight and a minor polypeptide of 45 kDa after analysis by SDS-PAGE (Venegas and Solari, 1995). Only the 50 KDa polypeptide had polymerase activity in a colorimetric activity in a gel technique. The purified enzyme was highly sensitive to inhibition by the dideoxythymidine triphosphate analog, and it is active in DNA synthesis using DNAase I-activated DNA as a template (Venegas and Solari, 1995). Also, a cDNA encoding for *T. cruzi* pol β was cloned by our group from a TcI lineage by using information from peptides obtained from the purification described earlier (Venegas et al., 2009). The cDNA encodes a protein of 403 amino acid residues, and it is similar to the one from a *T. cruzi* TcVI lineage (CL Brener), however, it differs in three amino acid residues in highly conserved segments of the polypeptide (Venegas et al., 2009). From the published sequence of *T. cruzi* CL Brener genome, a pol β-related polymerase was found, named pol β-PAK, since it shares a high homology amino acid sequence with *T. brucei* pol β-PAK, and they are related at the level of amino acid sequence with mammalian pol β (Venegas et al., 2009). The mRNA encoding pol β is highly expressed in both proliferative and non-proliferative developmental forms of *T. cruzi*, indicating that the enzyme performs important functions through the complete life cycle of the parasite (Venegas et al., 2009).

The cDNA encoding *T. cruzi* pol β from TcI and TcVI lineages have been cloned and expressed in a recombinant form from bacteria and their biochemical properties were described by Machado and colleagues (de Oliveira Lopes et al., 2008) and by our group (Maldonado et al., 2015). Also, a cDNA encoding pol β-PAK has been cloned and expressed in *E. coli* (de Oliveira Lopes et al., 2008) The biochemical and molecular functions of both pol β and pol β-PAK were compared (de Oliveira Lopes et al., 2008). Both have some similar molecular functions, but they differ in some others. The main functional characteristics of pol β and pol β-PAK will be summarized in the next section.

**Figure 4:**
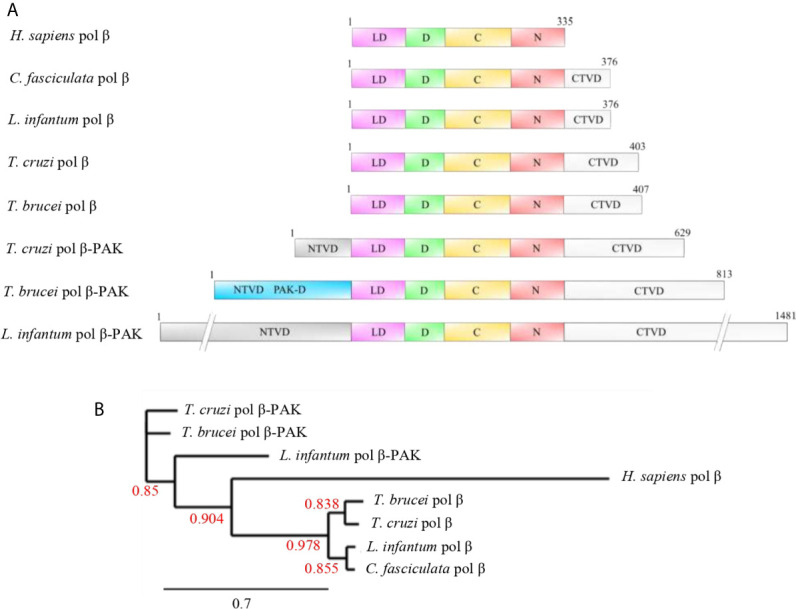
**(A)** Domain organization of selected pol β and pol β-PAK enzymes from different trypanosomatid species compared to human pol β. Pol β-PAKs are large polypeptides of variable length and contain N-terminal variable (NTVD; grey) and C-terminal variable (CTVD; white) domains with a central core, which contains the conserved lyase and polymerase domains. *T. cruzi* pol β-PAK contains the PAK-D (light blue), which is rich in Proline, Alanine, and Lysine residues. The functions of those variable domains are largely unknown, but we speculate that might contain modification sites to regulate protein function or might be engaged in species-specific protein-protein interactions. On the other hand, pols β are smaller polypeptides compared to pol β-PAK and contain the conserved lyase and polymerase domains to complement their biological role in BER and replication. They contain an extra short C-terminal domain, which most likely could modulate their biological activities. The lyase domain (LD; pink), D-subdomain (green), and C-N-subdomains (red). **(B)** Phylogenetic tree of the selected trypanosomatid pol β-like enzymes and human pol β. It can be observed that human pol β is closer to the trypanonosomatid pol β as they arose from the same ancestor, while pol β- PAK is rather distant compared to pol β. The Phylogenetic tree was constructed using the phylogeny.fr program and bootstrap values are given (red numbers). The bootstrap values show high confidence. The accession numbers of each polymerase are PBJ75947.1 (*T. Cruzi* pol β-PAK), AAQ56190.1 (*T. brucei* pol β-PAK), XP_001463421.1 (*L. infantum* pol β-PAK), NP_002681.1 (*H. sapiens* pol β), AAX79362.1 (*T. brucei* pol β), RNC61524.1 (*T. cruzi* pol β), AAF00495.1 (*L. infantum* pol β), and AAA68599.2 (*C. fasciculate* pol β). Dashes were introduced in the NTVD and CTVD in *L. infantum* pol β-PAK to fit into the figure.

As mentioned earlier, trypanosomatid pol β is multitalented and one of the smallest DNA polymerases belonging to the pol X family of DNA polymerases. This polymerase has two main domains, which can be defined based on the structure of the homolog human pol β. The N-terminus contains an 8 kDa domain with lyase activity, whereas the second domain contains three subdomains, namely, the DNA binding (D), catalytic (C), and nucleotide-binding (N) subdomains (Garcıa-Dıaz et al., 2001; Braithwaite et al., 2005; Prasad et al., 2017), which comprise the polymerase domain. Those domains allow pol β to perform its role in BER and to carry out other roles such as replication of the kDNA. The lyase domain can remove the 5’deoxyribose phosphate (dRP lyase) left after incision for the AP endonuclease during BER, and afterward the missing nucleotide is inserted by pol β itself and the nick can be sealed by a ligase. Also, the lyase domain of human Polβ participates in the processive search for DNA damage by interacting nonspecifically with DNA during the processive search process (Howard et al., 2017; Howard and Wilson, 2017; Howard et al., 2020). The functions of the other three subdomains are the binding to the DNA (D), template-guided incorporation of the missing nucleotide (C), and binding of the nucleotide for catalysis (N). The role of trypanosomatid pol β in kDNA replication and repair of oxidative lesions has been demonstrated in *T. cruzi* (Schamber-Reis et al., 2012; Rajao et al., 2014). On the other hand, it is most likely that pol β in *T. cruzi* is always bound to the DNA, even in the absence of DNA damage, as it can be crosslinked to kDNA (Rojas et al., 2018) and we speculate that carries out a similar role in processive searching as the lyase domain in human pol β. In *T. cruzi* pol β there is an extra domain, located at the C-terminus, which we named CK2-regulatory domain, since possesses several consensus sites for Casein Kinase 2 (CK2) phosphorylation, and perhaps this domain regulates its DNA synthesis activity. This domain is absent in human pol β, and it is shorter in *C. fasciculata* and *L. infantum* pol β, whereas only a single CK2 phosphorylation site is conserved in *T. brucei* pol β. The domain organization of those enzymes is described in **Figure 4A**. A phylogenetic analysis of the different polymerases is displayed in **Figure 4B**.

The *T. cruzi* pol β-PAK is larger than pol β and contains 629 amino acids, which can be divided into four domains (**Figure 4A**). A N-terminal variable domain (NTVD) and a second domain with dRP lyase activity. The third domain is the DNA polymerase and contains the three subdomains D-C-N and a fourth C-terminal extra domain (CTVD). Also, it has an insertion in between the dRP lyase domain and the DNA polymerase domain. The function of this insertion is currently unknown, however, is Glycine-rich and might function as a hinge region. The fourth CTVD extra domain, located at the C-terminal end of the polypeptide, contains phosphorylation consensus sites for Casein Kinase 1 (CK1) and Protein Kinase A (PKA). Both pol β and β-PAK have dRP lyase and DNA polymerase activities, however, only pol β-PAK possesses *in vitro* trans-lesion synthesis (TLS) activity, since it can perform DNA synthesis through an 8oxoG lesion (de Oliveira Lopes et al., 2008). *T. brucei* and *L. infantum* pol β-PAK are large polypeptides, however, their domain organization is similar to the other DNA polymerases displayed in **Figure 4A**.

Although, pol β and pol β-PAK locate at the mitochondria, they have different locations inside the kinetoplast, while pol β locates at the antipodal sites, pol β-PAK is located at the kDNA disk (Saxowsky et al., 2003; de Oliveira Lopes et al., 2008; Schamber-Reis et al., 2012). These observations indicate that the two enzymes perform distinct and non-redundant roles in kDNA repair and replication. The fact that pol β-PAK can replicate through 8oxoG lesions (de Oliveira Lopes et al., 2008) might indicate that this polymerase is more error-prone than pol β itself, and this feature could be a mechanism to generate mutations in the minicircles in sites away from the CBS to generate diverse gRNAs involved in mitochondrial mRNA editing.

The cellular functions of *T. cruzi* pol β have been studied by overexpressing the protein in epimastigote cells and then exposing the cells to high concentrations of genotoxic agents in the absence or presence of BER inhibitors. The results indicate that the overexpression of pol β protects the cells from the effects of those agents, and the presence of BER inhibitors greatly reduces the protective effects of the overexpression of pol β (Schamber-Reis et al., 2012). It has been shown by immunofluorescence studies that *T. cruzi* pol β is found at the antipodal sites of kDNA in the epimastigotes in the replication stage from G1/S until the early G2 phase (Schamber-Reis et al., 2012). However, in the late G2 phase, pol β spreads and it is no longer associated with the antipodal sites and this patter is maintained until the end of mitosis. Pol β is also located at the antipodal sites in amastigote developmental stages. Moreover, pol β is found in the mitochondrial matrix of trypomastigote cells (Schamber-Reis et al., 2012). When pol β is overexpressed in parasites, the levels of 8oxoG are reduced compared to normal cells (Schamber-Reis et al., 2012). Also, those pol β overexpressing-parasites showed increased resistance to hydrogen peroxide treatment, when compared with normal parasites (Schamber-Reis et al., 2012). Treatment of the pol β-overexpressing parasites with methoxyamine, which reacts to the apurinic/apyrimidinic sites inhibiting BER, prevents resistance to hydrogen peroxide treatment (Schamber-Reis et al., 2012). When epimastigote cells are treated with hydrogen peroxide, pol β forms a third focus in the kDNA in addition to those located at the antipodal sites (Schamber-Reis et al., 2012). Overexpression of pol β in epimastigote cells confers resistance to the drug benznidazole, which is one of the drugs used to treat patients with Chagas disease (Rajao et al., 2014). Since pol β is located into the mitochondria of *T. cruzi* cells, those observations indicate that benznidazole also induces damage to the kDNA (Rajao et al., 2014). We recently showed that *T. cruzi* epimastigote and trypomastigote cells exposed to hydrogen peroxide during short periods of time showed increased levels of pol β enzyme and two forms of the polymerase were identified and overexpressed after treatment with hydrogen peroxide (Rojas et al., 2018). The smaller one (L) was unphosphorylated, whereas the bigger one (H) was phosphorylated and active in DNA synthesis. Those results indicate that *in vivo* pol β must be phosphorylated to perform its function and is overexpressed in response to a genotoxic insult. pol β is associated physically with kDNA since it can be crosslinked to the kDNA; however, it is not associated with nuclear DNA (Rojas et al., 2018).

In both *T. cruzi* and *T. brucei*, pol β and pol β-PAK are nuclear-encoded enzymes, however, they are located in the mitochondrion (Saxowsky et al., 2003; Schamber-Reis et al., 2012). Also, in *C. fasciculata* pol β locates at the mitochondria, however, in other *Crithidia* species a pol β or pol β-PAK homologs cannot be found using BLAST searches. This indicates that a pol β encoding gene was deleted in the other *Crithidia* species during the speciation process or alternatively a pol β encoding gene was transferred from another trypanosomatid species into the *C. fasciculata* genome. Interestingly enough, in *Leishmania* species, the pol β enzyme is nuclear-located (Taladriz et al., 2001), however, a pol β-PAK encoding gene is found in the genome of these species. The pol β-PAK in *Leishmania* species is predicted to locate at the mitochondria; however, a biochemical characterization of this enzyme has not been done yet. This pol β-PAK homologue gene could fulfill the function of both pol β and pol β-PAK in the mitochondria of *Leishmania* species.

It is clear the role of pol β in *T. cruzi* and in other trypanosomatids is the kDNA replication and the repair, *via* BER, of oxidative DNA damage, which is produced by genotoxic agents, such as hydrogen peroxide and benznidazole. It is still unknown whether trypanosomatid pol β can repair short or long patches of DNA and the processivity of this polymerase is largely unknown. Also, the signals that regulate the levels and activity of pol β remain to be studied.

## Key Features of Trypanosomatid pol β Structure

As noted earlier, mammalian pol β has two enzymatic activities that contribute to the BER process. The activities are contained in two domains: the N-terminal 8 kDa lyase domain and the 31 kDa C-terminal polymerase domain. Trypanosomatid pol β display a similar domain structure, although the *T. cruzi* enzyme contains an extra CTVD domain which is rich in protein kinase CK2 phosphorylation sites, however, only one CK2 phosphorylation site is conserved in the *T. brucei* pol β.

**Figure 5:**
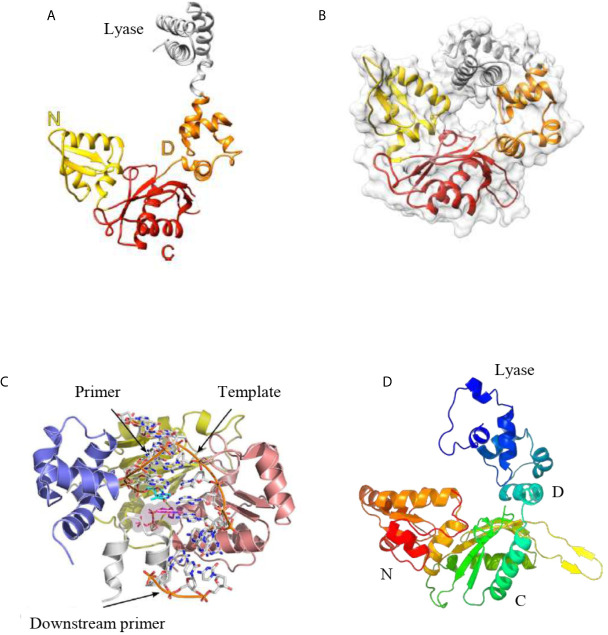
**(A)** Tridimensional structure of the human pol β apoenzyme. The apoenzyme can form an extended structure, in which the lyase domain and polymerase subdomains can be seen. **(B)** The tridimensional structure of human pol β in a ternary complex displays a doughnut-like structure in which the lyase domain and the N-subdomain interact. **(C)** The crystal structure of *L. infantum* pol β in the ternary complex adopts a similar structure as the human pol β in the ternary complex. **(D)**. Predicted tridimensional structure of *T. cruzi* pol β. The overall fold of this polymerase is similar to the structure of the human pol β apoenzyme **(A)**. The modeling was performed with the Phyre2 Program. In **(A, D)**, the domains are shown. Figures **(A–C)** were reproduced from references Mejia et al., 2014 and Beard and Wilson, 2014.

The tridimensional structure of mammalian pol β has been solved with a variety of substrates, which has provided a wealth of structural information. For over 40 years, Wilson and colleagues have studied mammalian pol β at the biological and molecular level (for a review see reference Whitaker and Freudenthal, 2020). Structures of mammalian pol β and others derived from the pol X family of polymerases indicate that they have the same modular domain organization (Yamtich and Sweasy, 2010; Bienstock et al., 2014). Those members involved in DNA repair contain the N-terminal lyase domain and the polymerase domain, which is composed of three functionally different subdomains, including the DNA-binding (D), catalytic (C), and nucleotide-binding (N) subdomains (**Figure 5A**). The D subdomain is involved in the proper positioning of the template base, whereas the C subdomain coordinates two divalent metal cations and is responsible for the DNA synthesis. The N subdomain is involved in the binding of the deoxynucleotide triphosphate substrate. The D and N subdomains are spatially located on opposite sides of the C subdomain in the apoenzyme (**Figure 5A**), however, in the presence of substrates, the tridimensional structure shows that the lyase domain physically interacts with the N subdomain (Yamtich and Sweasy, 2010; Beard and Wilson, 2019) (**Figure 5B**).

As noted earlier, usually trypanosomatids possesses more than one pol β-like enzymes. These enzymes are mitochondria-located in most of them, except in *Leishmania* species, where pol β is localized to the nucleus. Unfortunately, only one pol β crystal structure from trypanosomatids has been reported and derives from *Leishmania infantum* pol β, which is in a complex with a single-nucleotide gap substrate and a dNTP (Mejia et al., 2014). This crystal structure could shed light on the structure of *T. cruzi* and *T. brucei* pol β since they are highly conserved (70% sequence identity). The structural characterization reveals that *L. infantum* pol β conserves the overall conserved domain organization of pol X family of enzymes interacting with DNA in a similar manner to human pol β (Beard and Wilson, 2014) (**Figure 5C**). Modeling of the tridimensional structure of *T. cruzi* pol β indicates that adopts the common pol X family fold as it is expected (**Figure 5D**). The overall predicted structure is similar to *L. infantum* and human pol β. It is important to state that family-X polymerases contain variable regions in between β-strands, which can form variable loops (Delarue et al., 2002; Mejia et al., 2014; Beard and Wilson, 2014). These loops are important to the pol X family of polymerases to perform specific functions. In *L. infantum* pol β, there is a variable loop at the C-terminus (loop 3) which interacts with the template strand upstream of the nascent base pair (Mejia et al., 2014). The deletion of this loop results in the higher catalytic activity of the *L. infantum* DNA pol β (Mejia et al., 2014). In *T. cruzi* pol β, this loop is longer as compared to the *L. infantum* enzyme and most likely plays a similar role, besides that contains several protein kinase CK2 phosphorylation sites.

**Figure 6:**
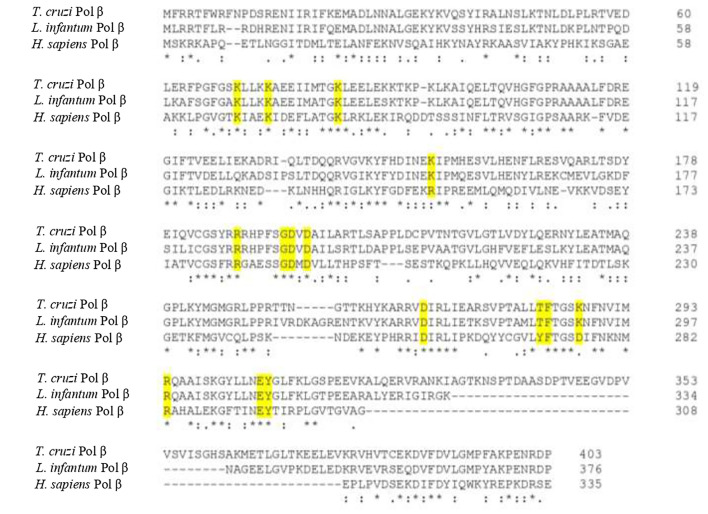
Protein sequence alignment of *T. cruzi, L. infantum*, and human pol β shows the key conserved residues involved in BER (yellow shaded). Those residues are also conserved in other trypanosomatid pol β enzymes as well. The alignment was done using the Clustal Omega program.

We have done an extensive comparative analysis of the amino acid residues critical for pol β function and it shows that most of them are conserved in both *T. cruzi* and *L. infantum* pol β with respect to human DNA pol β (Yamtich and Sweasy, 2010; Bienstock et al., 2014; Mejia et al., 2014; Beard and Wilson, 2014). Those key conserved residues are shown in **Figure 6**. The dRP lyase nucleophile Lys72 of human pol β is conserved in *T. cruzi* (Lys74) and *L. infantum* (Lys72) enzymes and Lys68, which corresponds to Lys70 in *T. cruzi* pol β and Lys68 in the *L. infantum* enzyme. Another conserved residue is Lys84 of human pol β, which corresponds to Lys86 in *T. cruzi* DNA pol β and Lys84 in pol β from *L. infantum*. Lys68 is important for the binding of the 5’-PO4 (5’-dRP) moiety of the gap and Lys72 acts as the primary amine forming a Schiff-base intermediate for excision of the 5’-dRP moiety during the BER process. Lys84 acts as an alternative nucleophile in human pol β and most likely has the same function in trypanosomatid pol β. The 8-kDa lyase domain of *L. infantum* pol β is engaged in the binding of the 5’-PO4 of the gapped DNA, however, the tridimensional structure of this domain was not completely resolved due to disorder in the structure of the lyase domain (Mejia et al., 2014).

The active site of *L. infantum* pol β shows the same structural features as those observed in human DNA pol β (Yamtich and Sweasy, 2010; Bienstock et al., 2014; Beard and Wilson, 2014). The active site is the DXD motif, which corresponds to Asp190 and Asp192 in human pol β and to Asp194 and Asp196 in the *L. infantum* enzyme. In *T. cruzi* pol β, those catalytic residues correspond to Asp195 and Asp197. The other important catalytic residue in human pol β is Asp256, which corresponds to Asp271 in the *L. infantum* enzyme and Asp267 in *T. cruzi* pol β. In human pol β Asp192 can coordinate two active-site Mg2+ metal ions required for DNA synthesis (Yamtich and Sweasy, 2010; Bienstock et al., 2014; Beard and Wilson, 2014). Also, Arg283 in the human polymerase is conserved as is key for fidelity since it functions in the proper positioning of the templating base and corresponds to Arg298 in *L. infantum* pol β and to Arg294 in the *T. cruzi* enzyme.

Residues important for dNTP binding of human pol β are in part contained in the YFTGSDIFNK motif (residues 271-280; 38,70), whereas in *T. cruzi* and *L. infantum* pol β is TFTGSKNFNV (residues 286-295 and 282-291 in the *T. cruzi* enzyme). That motif contains Asp276, which forms hydrogen bonds with O3’ of the incoming nucleotide. Other important residues are Arg183, which coordinates non-bridging oxygens on the α-phosphate (Pα) and β-phosphate (Pβ) of the incoming nucleotide, while Arg149 and Gly189 coordinate the γ-phosphate (Pγ) of the incoming nucleotide. Asp276 in the human enzyme is substituted by Lys291 in *L. infantum* pol β and by Lys287 in the *T. cruzi* polymerase. Arg183 is conserved in *L. infantum* pol β (Arg187) and in the *T. cruzi* enzyme (Arg188). Gly189 in the human enzyme is also conserved and correspond to Gly193 in the *L. infantum* polymerase and to Gly194 in *T. cruzi* pol β, while Arg149 is replaced by Lys152 in *L. infantum* and for Lys153 in *T. cruzi* pol β. A detailed tridimensional structure of *L. infantum* pol β in a ternary complex (enzyme-DNA template-nucleotide) can be found in the paper by Mejia et al. (2014).

**Figure 7:**
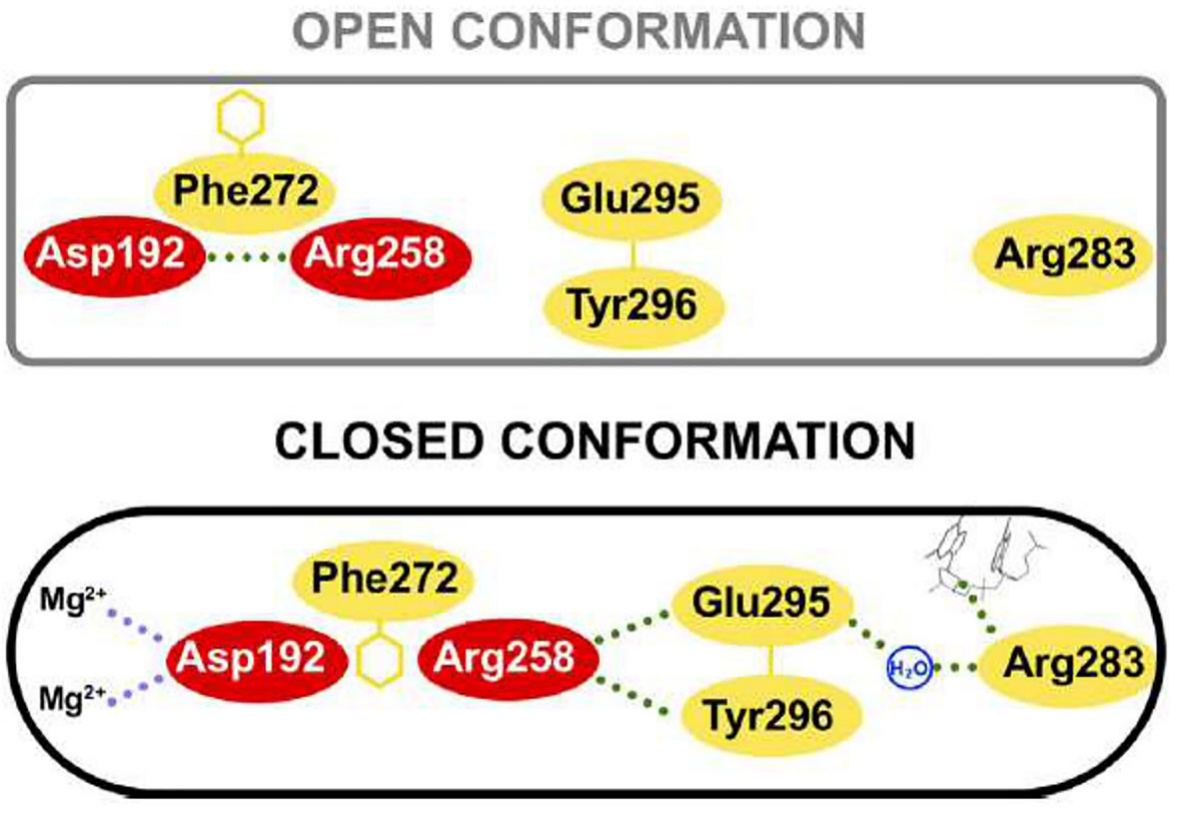
Key residue interactions during the open and closed conformation of human pol β. In the open conformation Arg258 does not interact with Glu295 and Tyr296, however, in the closed conformation, Arg558 can interact with those residues and Glu295 can interact with Arg283, which in turn interacts with the templating coding base. Also, the closed conformation can coordinate two Mg2+ metal ions. Residues of the C-subdomain are red. This figure was reproduced from reference Beard and Wilson, 2014.

The structure of human pol β in the ternary complex is in a doughnut-like structure (**Figure 5B**), where the N-terminal lyase domain interacts with the N-subdomain of the polymerase domain and those interactions are key during the processing of the 5’ and 3’-ends of the gapped DNA during BER (Beard and Wilson, 2014; Beard and Wilson, 2019). When it is compared the binary complex (enzyme-DNA template) with the ternary complex (enzyme-DNA template-dNTP) it is seen that the N-subdomain repositions itself and closes around the nascent base pair on the DNA (Beard and Wilson, 2014; Beard and Wilson, 2019). The lyase domain also closes and the interactions between the lyase domain and N-subdomain are enhanced, stabilizing a closed conformation. The transition from an open binary complex to a closed ternary complex can induce changes in the local hydrogen bonding involving a critical Asp192 of the catalytic site, which is required to coordinate two Mg2+´metal ions involved in DNA synthesis (**Figure 7**; Beard and Wilson, 2014; Beard and Wilson, 2019). The transition from an open to a closed form is induced by the binding of dNTP-Mg2+, since the binding of a dNTP without a coordinating metal does not induce a conformational change. During the open to closed transition state, an altered hydrogen bonding interaction occurs between key residues of the D and C-subdomains (Beard and Wilson, 2014; Beard and Wilson, 2019). Those changes involve Asp192, Arg258, Phe272, Glu295, Tyr296, and Arg283. In the open conformation, Asp192 interacts with Arg258; however, in the closed conformation, the interaction between these two residues is interfered by Phe272, and Arg258 can now interact with Glu295 and Tyr296, which in turn can interact with Arg283, which can interact with the template strand. Therefore, the position of the N-subdomain can be structurally transmitted to the active catalytic site and enables Asp192 to coordinate two Mg2+ metal ions (**Figure 7**; Beard and Wilson, 2014; Beard and Wilson, 2019). As mentioned earlier, Asp192 and Arg258 from human pol β are conserved in trypanosomatid pol β and the rest of the residues are also conserved as well, including Phe272 (Phe282 in *T. cruzi* and Phe287 in *L. infantum* pol β), Glu295 (Glu306 in *T. cruzi* and Glu310 in *L. infantum* pol β), and Tyr296 (Tyr307 in *T. cruzi* and Tyr311 in the *L. infantum* enzyme). Arg283 of human pol β is also conserved in the tripanonosomatid DNA pol β and corresponds to Arg294 in *T. cruzi* pol β and Arg298 in the *L. Infantum* polymerase.

## Concluding Remarks

The minicircle kDNA replication is an extremely complex process that requires several proteins to be completed. Moreover, the architecture of the kDNA is unusual, and replication proteins are located at discrete domains into the kinetoplast. We expect that many other components of the replication machinery will be uncovered and the functions of those components studied. It is still unknown how the sister minicircles are moved to the antipodal sites and which are the motors and the mechanisms. Unfortunately, *in vitro* systems for kDNA replication and kDNA repair have not been developed yet, perhaps due to the complexity of the whole process. Studies using a biochemical assay would provide many answers, such as the minimal components required for minicircle kDNA replication and the function of each component.

The pol β-like enzymes in trypanosomatids play a fundamental role in the repair of oxidative lesions in the mitochondria and kDNA replication as they are mitochondria-located in most trypanosomatids, apart from *L. infantum* and other *Leishmania* species. However, their biological functions have not been as deeply studied as their mammalian counterparts. It is probable that many other functions will be discovered in the future as biochemical and cellular studies can progress. Also, very little is known about the regulation of its activity, which most likely is regulated at the post-translational modification level, such as phosphorylation for example. This is another interesting field to investigate.

Structural and biological studies of trypanosomatid pol β-like enzymes are limited even though they are necessary for the development of new drugs to target this enzyme family since they are key players for parasite survival. Also, other proteins of the replication machinery or kDNA repair could be targeted to treat the different diseases caused by parasites of this order. Any attempt to design drugs against components of the parasite replication machinery will necessarily require structural and biological studies of those components. We expect that soon biological and structural studies can provide fundamental molecular details on kDNA repair and kDNA replication carried out by this polymerase family in trypanosomatids.

## Author Contributions

Both SM-P and FU performed the bibliographical search and wrote the first draft for the manuscript. EM and AS wrote the final manuscript. All authors contributed to the article and approved the submitted version.

## Funding

This work was funded by Grant FONDECYT 1190392 to AS and an internal Grant from the ICBM, Faculty of Medicine, the University of Chile to EM.

## Conflict of Interest

The authors declare that the research was conducted in the absence of any commercial or financial relationships that could be construed as a potential conflict of interest.

## Publisher’s Note

All claims expressed in this article are solely those of the authors and do not necessarily represent those of their affiliated organizations, or those of the publisher, the editors and the reviewers. Any product that may be evaluated in this article, or claim that may be made by its manufacturer, is not guaranteed or endorsed by the publisher.
